# Risk factors of postoperative complications after emergency repair of incarcerated groin hernia for adult patients: a retrospective cohort study

**DOI:** 10.1007/s10029-018-1854-5

**Published:** 2018-11-12

**Authors:** W. Dai, Z. Chen, J. Zuo, J. Tan, M. Tan, Y. Yuan

**Affiliations:** 1 Center of Gastrointestinal Surgery, The First Affiliated Hospital of Sun Yat-sen University, Guangzhou, 510080 Guangdong Province People’s Republic of China; 2 Center of Hernia and Abdominal Wall Surgery, The First Affiliated Hospital of Sun Yat-sen University, Guangzhou, 510080 Guangdong Province People’s Republic of China

**Keywords:** Incarcerated groin hernia, Herniorrhaphy, Synthetic mesh, Complications, Risk factors

## Abstract

**Purpose:**

This study aimed to explore risk factors of postoperative complications for adult patients with incarcerated groin hernia (IGH).

**Methods:**

From January 2010 to December 2017, consecutive patients undergoing emergency hernia repair for IGH in our center were retrospectively reviewed. Postoperative complications, such as surgical site infection, seroma, hernia recurrence and mortality, were investigated, with risk factors for such complications analyzed using univariate and multivariate regressions.

**Results:**

Sixty-four patients were included, with 51 males and 13 females (mean age 65.1, range 25–98 years). Ten patients (15.6%) underwent resection of necrotic bowel and anastomosis. 43 patients (67.2%) received open tension-free herniorrhaphy with polypropylene mesh, whereas the rest (32.8%) received herniorrhaphy without mesh. The overall postoperative complication rate was 40.6% (26/64), with an incisional complication rate of 31.2% (20/64) and an infection rate of 6.2% (4/64). At a median follow-up of 32 months, hernia recurrence and mortality were recorded in five cases each (7.8%). Mesh repair was associated with decreased recurrence rate compared with non-mesh repair (2.3% vs. 19.0%, *p* = 0.019). Diabetes mellitus (OR 8.611, 95%CI 1.292–57.405; *p* = 0.026) was an independent risk factor of postoperative complications, together with chronic obstructive pulmonary disease (COPD; OR 14.365, 95%CI 1.652–127.767, *p* = 0.016), intestinal necrosis (OR 14.260, 95%CI 1.079–188.460, *p* = 0.044), and general anesthesia (OR 14.543, 95%CI 1.682–125.711, *p* = 0.015) as risk for incisional complications after surgery.

**Conclusions:**

Diabetes mellitus was an independent risk factor of postoperative complications for IGH, along with COPD, intestinal necrosis and general anesthesia associated with incisional complications. The use of polypropylene mesh did not increase infection or recurrence rate in this cohort.

## Introduction

Incarcerated abdominal wall hernia, defined as the inability to reduce the hernia content from abdominal wall, is a common surgical emergency, which accounts for 5–15% of abdominal hernias. Incarcerated groin hernia (IGH) is one of the most frequently encountered types, accounting for 50–80% of incarcerated abdominal hernias. About 15% of IGH patients require bowel resection due to progressive bowel necrosis [1–4]. The incidence of postoperative complications in emergency repair of IGH is 21–39%, along with a mortality rate of 4–5% [1–4]. Early recognition of risk factors for postoperative complications and rapid effective interventions for potential complications are of great clinical significance.

The application of synthetic mesh in emergency surgery for IGH remains controversial, especially when the presence of bowel necrosis, contaminated or infected surgical field is clinically validated [5]. Recently, several studies have revealed that synthetic mesh could be safely and effectively used in patients with incarcerated or strangulated inguinal hernia, which significantly reduced the risk of hernia recurrence but did not increase the opportunity of surgical site infection (SSI) [1, 2, 4–8]. Nevertheless, most of those studies are retrospective analyses, and the level of evidence-based surgical practice is not high enough. As a result, more evidence-based clinical data are necessary to confirm the safety and effectiveness of using synthetic mesh in repairing IGH.

The purpose of this study was twofold: first, to investigate risk factors of postoperative complications for patients with IGH; and second, to evaluate the safety and efficacy of clinical usage of synthetic mesh in emergency herniorrhaphy for the IGH cohort.

## Methods

From January 2010 to December 2017, consecutive adult patients who had confirmed IGH diagnosis and underwent emergency herniorrhaphy in our center were retrospectively reviewed. All data were retracted from medical records and our database of outpatient visits during the follow-up period. Patients who died from their comorbidities and those who were lost to follow-up were included for the final analysis. The study protocol was approved by the Institute Review Board of the ethical committee of our hospital, with written informed content waived due to its retrospective design.

The synthetic mesh used in emergency herniorrhaphy for all patients was polypropylene (PP) mesh, which was made of light-weight (40 g/m^2^) and large-pore (3 mm) PP patch, and monofilament-braided wire (0.13 mm). This non-absorbable mesh was commonly applied in open inguinal herniorrhaphy, with reduced incidence of chronic groin pain as well as other groin symptoms [9].

### Perioperative management

Immediately after admission, antibiotic prophylaxis and fluid resuscitation were initiated against underlying infection and water–electrolyte imbalance. For patients with intestinal obstruction, *nil per os* and nasogastric tube decompression were kept before surgery. The emergency operation was basically decided by the main surgeons, according to their clinical judgment and preference. The administration of antibiotics would be extended for 5–7 days when bowel resection was performed due to confirmed necrosis, perforation, or severe surgical site contaminations. Additionally, surgical drainage and therapeutic antibiotic treatment according to bacteriologic culture results were continued for at least 7 days, when specific complications such as incisional and abdominal infection occurred.

During surgery, incarcerated hernia was repaired with various techniques. Briefly, as for IGH without ischemic necrosis of the hernia content, a simple reduction of the hernia content was applied, followed by a high ligation of hernia sac at the level of internal inguinal ring. After that, PP mesh was used to achieve tension-free repair when the Lichtenstein procedure was considered for inguinal hernia, with non-mesh tissue repair performed when Bassini or Shouldice procedure was considered [10]. The mesh-plug repair was employed for femoral hernia, with non-mesh tissue repair performed when the McVay procedure was preferred [11].

**Fig. 1:**
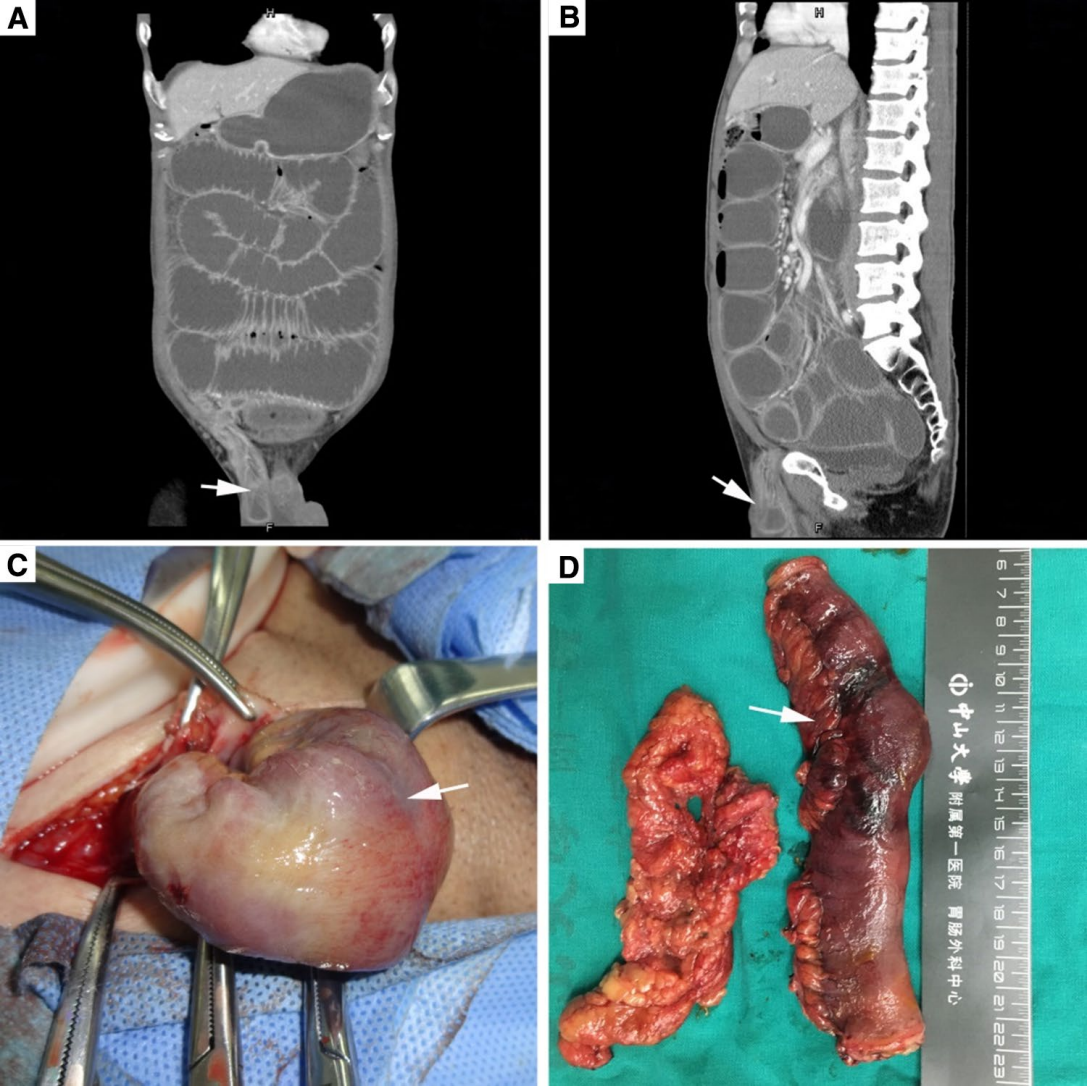
Demonstration of surgical management of incarcerated groin hernia. This was a 46-year-old male patient who presented with severe groin pain and abdominal distension for 10 h. **a** Coronal view of abdominal CT scan; **b** sagittal view of abdominal ct scan; **c** surgical exploration from the groin incision; **d** resected bowel and greater omentum strangulated in the right groin area. The white arrows in both **a** and **b** show the strangulated inguinal hernia on the right-sided groin. In addition, the patient has intestinal obstruction. White arrow heads (**c** and **d**) refer to intestinal necrosis

As for IGH with bowel necrosis but no perforation, a resection of necrotic bowel plus extracorporeal anastomosis via an inguinal incision was performed, as illustrated in Fig. 1. Afterward, a high ligation of hernia sac was performed, followed by a mesh or tissue repair mainly determined by surgeons. As for IGH with bowel perforation, a laparotomy from a mid-line incision would be made, followed by a simultaneous bowel resection and a high ligation of hernia sac.

A closed non-suction drainage system was supplied to the surgical wound once an enterectomy performed or contaminated wound confirmed during surgery. Of note, the end of inserted drain should be placed into peritoneal cavity in specific cases with bowel necrosis or peritonitis. The drain was removed after the drainage had decreased to 50 ml/day.

After discharge, patients were scheduled to regular outpatient visits at least for a 1-year follow-up period. During these visits, physical examination, abdominal ultrasound, particularly CT scans were performed to determine hernia recurrence and other long-term complications. The end date of follow-up was March 31, 2018. A recorded death or recurrence was regarded as the endpoint of the study.

### Data collection and study outcomes

All included cases were reviewed by two researchers (DW and CZ) independently. Clinical data, including demographic characteristics (gender, age, concomitant disease, etc.) and basic information of IGH, were collected from the medical records and database meanwhile. Next, data related to postoperative complications were extracted and double checked by both researchers. Surgical wound classification was consistent with the Centers for Disease Control and Prevention (CDC) wound classification, as well as the diagnostic criteria of SSI [12].

The primary outcomes included postoperative complications within 30 days of surgery, hernia recurrence and long-term mortality during the follow-up period. Patients were then stratified into two groups based on mesh or non-mesh repair technique applied during surgery to evaluate the safety and efficacy of treatment options.

### Statistical analysis

Descriptive data were presented as mean ± standard deviation (SD) or median (range or 95% confidential interval [95%CI]) as appropriate. Chi-square test was used for the comparison of categorical variables. Student’s *t* test and Mann–Whitney *U* test were employed for comparisons of normal and non-normal continuous variables, respectively. Univariate and multivariate logistic regressions were used to explore risk factors of postoperative complications. All data analyses were performed using the SPSS for windows (version 23, IBM, Chicago, IL, USA). A two-tailed *p* value < 0.05 or an odds ratio (OR) with 95%CI not inclusive of the unity was considered significant.

## Results

Within the study period, a total of 64 patients were included, with 51 males (79.7%) and 13 females (20.3%), and an average age of 65.1 (range 25–98) years. The baseline characteristics of all subjects are summarized in Table 1. It showed that male was more involved than female, almost reaching up to 80% of cases. Besides, 57.8% of all patients were older than 65 years, and 62.5% of them had various concomitant diseases when admitting to our center.

**Table 1:** Baseline characteristics of patients with incarcerated groin hernia

Variables	The pooled (*N* = 64)	Mesh group (*N* = 43)	Non-mesh group (*N* = 21)	*p* value
Gender (male), *n* (%)	51 (79.7)	36 (83.7)	15 (71.4)	0.324
Age (years), mean ± SD	65.1 ± 17.1	62.5 ± 17.9	70.5 ± 14.1	0.078
≥ 65 years, *n* (%)	37 (57.8)	21 (48.8)	16 (76.2)	0.058
Concomitant disease conditions, *n* (%)	40 (62.5)	23 (53.5)	17 (81.0)	0.053
Diabetes mellitus	12 (18.8)	6 (14.0)	6 (28.6)	0.185
Hypertension	33 (51.6)	18 (41.9)	15 (71.4)	0.035*
Heart diseases	15 (23.4)	9 (20.9)	6 (28.6)	0.540
COPD	14 (21.9)	7 (16.3)	7 (33.3)	0.196
Chronic nephropathy/renal failure	2 (3.1)	1 (2.3)	1 (4.8)	1.000
Duration of hernia (years), *M* (95%CI)	5.0 (5.4–12.0)	5 (3.4–6.0)	7 (2.3–10.0)	0.891
Duration of hernia incarceration (h), *M* (95%CI)	24.0 (32.4–52.5)	23 (13.0–48.0)	25 (14.7–48.0)	0.365
Maximum diameter of hernia sac (cm), *M* (95%CI)	7 (6.9–8.8)	6 (5.8–8.0)	8 (5.8–13.3)	0.117
Bowel necrosis, *n* (%)	10 (15.6)	1 (2.3)	9 (42.9)	< 0.001*
Bowel perforation, *n* (%)	2 (3.1)	0	2 (9.5)	0.104
Preoperative intestinal obstruction, *n* (%)	22 (34.4)	9 (20.9)	13 (61.9)	0.002*
Abdominal wall hernia history, *n* (%)	4 (6.2)			
Abdominal surgery history, *n* (%)	6 (9.4)	4 (9.3)	2 (9.5)	1.000
Classification of groin hernia, *n* (%)				0.020*
Indirect hernia	47 (73.4)	34 (79.1)	13 (61.9)	
Direct hernia	5 (7.8)	5 (11.6)	0	
Femoral hernia	11 (17.2)	4 (9.3)	7 (33.3)	
Saddle-bag hernia	1 (1.6)	0	1 (4.8)	
Recurrent hernia, *n* (%)	3 (4.7)	3 (7.0)	0	0.545
Scrotal hernia, *n* (%)	43 (84.3)	30 (83.3)	13 (86.7)	1.000
Gilbert–Rutkow type, *n* (%)				0.039*
II	5 (7.8)	5 (11.6)	0	
III	42 (65.6)	29 (67.4)	13 (61.9)	
VI	4 (6.2)	4 (9.3)	0	
V	1 (1.6)	1 (2.3)	0	
VI	1 (1.6)	0	1 (4.8)	
VII	11 (17.2)	4 (9.3)	7 (33.3)	
Hernia location (right sided), *n* (%)	40 (62.5)	23 (53.5)	17 (81.0)	0.053

*SD* standard deviation, *M* median, *CI* confidence interval, *COPD* chronic obstructive pulmonary disease

*Scrotal hernia* defined as hernia extending into the scrotum, Saddle-bag hernia defined as a combination of indirect and direct sacs on both sides of the inferior epigastric vessels, *Statistically significant

The median duration of groin hernia for this cohort was 60 (range 1–70) months. The median time interval until diagnosis of IGH was 24 (range 2–168) hours. The median size of hernia sac was 7 (range 3–18) cm in diameter. Bowel necrosis was recorded in 10 cases (15.6%), with intestinal perforation in 2 cases (3.1%) and intestinal obstruction in 22 cases (34.4%). IGH occurred more frequently in the right-sided groin than in the left-sided groin [62.5% (40/64) vs. 37.5% (24/64)], and the rate of bowel necrosis in femoral hernia was significantly higher than that in inguinal hernia [36.3% (4/11) vs. 11.3% (6/53), *p* = 0.037].

### Surgical data

Emergency herniorrhaphy was successfully completed in all patients, with no postoperative death or readmission observed until 30 days after discharge. Open tension-free herniorrhaphy was performed in 43 cases (67.2%), with non-mesh herniorrhaphy applied in the rest of 21 cases (32.8%). Laparoscopic herniorrhaphy was not recorded. The detailed surgical information is shown in Table 2. Briefly, the median operation time was 95 (range 50–180) min. Bowel resection and anastomosis were performed in ten cases (15.6%). Among those ten patients, one patient received PP mesh repair after a resection of necrotic bowel since the surgical field was presumed clean. The remaining nine patients underwent non-mesh tissue repair alone after bowel resection.

**Table 2:** Perioperative data of patients with incarcerated groin hernia

Variables	Value
ASA grades, *n* (%)	
Grade I	23 (35.9)
Grade II	28 (43.8)
Grade III	13 (20.3)
Anesthetic methods, *n* (%)	
Local	4 (6.2)
Spinal	32 (50.0)
General	28 (43.8)
Operation time (min), M (95%CI)	95 (91.9–107.8)
Open tension-free herniorrhaphy, *n* (%)	43 (67.2)
Lichtenstein	26 (40.6)
Mesh-plug repair	10 (15.6)
Preperitoneal herniorrhaphy	7 (10.9)
Traditional herniorrhaphy, *n* (%)	21 (32.8)
High ligation of hernia sac	4 (6.2)
Laparotomy plus high ligation	2 (3.1)
Bassini method	8 (1.25)
McVay method	6 (9.37)
Shouldice method	1 (1.56)
Postoperative LOS (days), *M* (95%)	5.0 (5.3–7.9)
Enterectomy, *n* (%)	10 (15.6)
Small intestine	9 (14.06)
Caecum	1 (1.56)

*M* median, *CI* confidence interval, *ASA* American society of anesthesia score, LOS length of stay

### Postoperative complications and long-term outcomes

The overall postoperative complication rate was 40.6% (26/64), and the incisional complication rate was 31.2% (20/64). Local wound complications were much more commonly observed than other complications (Table 3). Of note, one case repaired with PP mesh developed mild mesh infection after 6 days of surgery; however, the mesh was not retrieved from the surgical site after an effective wound healing process using a negative pressure drainage system. No additional surgery was appended for those complications according to data collected from medical records.

**Table 3:** Postoperative complications and follow-up results

Variables	Value
Postoperative complications, *n* (%)	26 (40.6)
Incisional complications	20 (31.2)
Seroma	19 (29.7)
Incisional infection	4 (6.2)
Scrotal swelling	13 (27.1)
Retention of urine	6 (9.4)
Intra-abdominal infection	1 (1.5)
Acute heart failure/arrhythmia	2 (3.1)
Pulmonary infection	1 (1.5)
Mesh infection	1 (2.3%)
Mesh extraction	0 (0)
Postoperative follow-up duration (months), *M* (95%CI)	32 (31.6–43.8)
Access to follow-up cases, *n* (%)	57 (89.1)
Hernia recurrence, *n* (%)	5 (7.8)
Hernia unrelated death, *n* (%)	5 (7.8)

*M* median, *CI* confidence interval

At the end time of follow-up, 57 of the 64 patients (89.1%) had complete follow-up records, with a median follow-up period of 32 (range, 31.6–43.8) months. Briefly, two patients (3.1%) lost contact with us, and five patients (7.8%) died. Of the five dead cases, two died of heart disease, two of multiple organ failure and one of respiratory failure. Hernia-related death was not observed, but hernia recurrence after initial herniorrhaphy was recorded in five cases (7.8%). All relapsed hernias were successfully managed with second operations.

### Mesh versus non-mesh repair

To evaluate the safety and efficacy of mesh repair for IGH treatment, all patients were divided into two groups: 43 cases (67.2%) and 21 cases (32.8%) in the mesh and non-mesh repair groups, respectively (Table 4). The average operation time in the mesh group was much shorter than that in the non-mesh group (87.4 min vs. 125.5 min, *p* < 0.001). Importantly, the incidence of overall postoperative complication (27.9% vs. 66.7%, *p* = 0.005), incisional seroma (20.9% vs. 47.6%, *p* = 0.028) and scrotal swelling (13.9% vs. 66.7%, *p* = 0.001), and median postoperative length of stay (5 vs. 8 days, *p* = 0.026) were markedly decreased in the mesh repair group compared with the non-mesh repair group. Besides, the hernia recurrence rate was also significantly reduced (2.3% vs. 19.1%, *p* = 0.019), with a comparable mortality rate observed between both groups (7.0% vs. 9.5%, *p* = 0.721).

**Table 4:** Comparisons of perioperative data between mesh repair group and non-mesh repair group

Variables	Mesh repair group (*n* = 43)	Non-mesh repair group (*n* = 21)	*p* value
Anesthetic methods, *n* (%)			0.001*
Local	3 (7.0)	1 (4.8)	
Spinal	28 (65.1)	4 (19.0)	
General	12 (27.9)	16 (76.2)	
Operation time (min), mean ± SD	87.4 ± 17.8	125.5 ± 38.5	< 0.001*
Enterectomy, *n* (%)	1 (2.3)	9 (42.9)	< 0.001*
Postoperative complications, *n* (%)	12 (27.9)	14 (66.7)	0.003*
Incisional seroma	9 (20.9)	10 (47.6)	0.028*
Incisional infection	1 (2.3)	3 (14.3)	0.099
Scrotal swelling	5 (11.6)	8 (38.1)	0.001*
Mesh infection	1 (2.3)	0	NA
Urine retention	3 (7.0)	3 (14.3)	0.385
Postoperative LOS (days), *M* (95%CI)	5 (3.8–6.0)	8 (5.0–8.0)	0.026*
Follow-up period (months), *M* (95%CI)	32 (24.5–46.8)	32 (22.4–53.2)	0.672
Recurrence rate, % (n/N)	2.3 (1/43)	19.0 (4/21)	0.019*
Overall mortality rate, % (n/N)	7.0 (3/43)	9.5 (2/21)	0.721

*NA* not available, *M* median value, *CI* confidential interval, *SD* standard deviation, *LOS* length of stay, *N* total number

*Statistically significant

### Risk factors of postoperative complications

The univariate regression analysis indicated that concomitant basic disease (*p* = 0.049), diabetes mellitus (DM, *p* = 0.001), chronic heart disease (CHD, *p* = 0.019), history of abdominal wall hernia (*p* = 0.024), bowel necrosis (*p* = 0.011), general anesthesia (*p* = 0.009), and mesh repair (*p* = 0.003) were risk factors of postoperative complications for IGH patients (Table 5). Specifically, concomitant basic disease (*p* = 0.002), DM (*p* < 0.001), hypertension (*p* = 0.047), CHD (*p* = 0.010), chronic obstructive pulmonary disease (COPD, *p* = 0.001), bowel necrosis (*p* = 0.001), general anesthesia (*p* = 0.016), and mesh repair (*p* = 0.028) were risk factors for postoperative incisional complications.

**Table 5:** Univariate regression analysis of risk factors of postoperative complications for incarcerated groin hernia

Variables	Postoperative complications			Incisional complications		
	Control (*N* = 38)	Event (*N* = 26)	*p* value	Control (*N* = 44)	Event (*N* = 20)	*p* value
Gender (male/female), *n* (%)	31 (81.6)/7 (18.4)	20 (76.9)/6 (23.1)	0.649	38 (86.4)/6 (13.6)	13 (65.0)/7 (35.0)	0.090
Age (≥ 65 years), *n* (%)	19 (50.0)	18 (69.2)	0.126	22 (50.0)	15 (75.0)	0.061
Concomitant basic disease, *n* (%)	20 (52.6)	20 (76.9)	0.049*	22 (50.0)	18 (90.0)	0.002*
Diabetes mellitus	2 (5.3)	10 (38.5)	0.001*	2 (4.6)	10 (50.0)	< 0.001*
Hypertension	17 (44.7)	16 (61.5)	0.187	19 (43.2)	14 (70.0)	0.047*
Heart disease	5 (13.2)	10 (38.5)	0.019*	6 (13.6)	9 (45.0)	0.010*
COPD	6 (15.8)	8 (30.8)	0.155	4 (9.1)	10 (50.0)	0.001*
Chronic kidney disease	1 (2.6)	1 (3.8)	1.000	2 (4.6)	0	1.000
Scrotal hernia, *n* (%)	26 (68.4)	17 (65.4)	1.000	30 (68.2)	13 (65.0)	0.419
Duration of hernia (years), *M* (95%CI)	5 (3.0–6.5)	5.5 (2.0–10.0)	0.431	5 (4.7–7.5)	4 (1.8–10.0)	0.599
Duration ≥ 5 years, *n* (%)	23 (60.5)	15 (57.7)	0.821	28 (63.6)	10 (50.0)	0.303
Duration of incarceration (h), *M* (95%CI)	23 (12.0–48.0)	36 (18.0–80.0)	0.115	23 (18.0–48.0)	23.5 (12.0–63.7)	0.738
Duration ≥ 24 h, *n* (%)	17 (44.7)	15 (57.7)	0.309	21 (47.7)	11 (55.0)	0.590
History of abdominal wall hernia, *n* (%)	0	4 (15.4)	0.024*	2 (4.6)	2 (10.0)	0.583
History of abdominal surgery, *n* (%)	3 (7.9)	3 (11.5)	0.680	3 (6.8)	3 (15.0)	0.366
Maximum diameter of hernia sac (cm), *M* (95%CI)	6 (5.0–8.0)	8 (5.0–10.0)	0.123	6.5 (5.0–8.0)	9 (5.0–12.0)	0.161
Diameter ≥ 10 cm, *n* (%)	10 (26.3)	12 (46.1)	0.101	12 (27.3)	10 (50.0)	0.076
Intestinal necrosis, *n* (%)	2 (5.3)	8 (30.8)	0.011*	2 (4.6)	8 (40.0)	0.001*
Intestinal perforation, *n* (%)	0	2 (7.7)	0.161	0	2 (10.0)	0.094
Concomitant intestinal obstruction, *n* (%)	11 (28.9)	11 (42.3)	0.269	13 (29.6)	9 (45.0)	0.228
Classification of hernia, *n* (%)			0.476			0.283
Indirect inguinal hernia	27 (71.0)	20 (76.9)		34 (77.3)	13 (65.0)	
Direct hernia	4 (10.5)	1 (3.8)		4 (9.1)	1 (5.0)	
Femoral hernia	7 (18.4)	4 (15.4)		6 (13.6)	5 (25.0)	
Saddle-bag hernia	0	4 (15.4)		0	1 (5.0)	
Recurrent hernia, *n* (%)	1 (2.6)	2 (7.7)	0.561	1 (2.3)	2 (10.0)	0.228
Hernia location (right-sided), *n* (%)	23 (60.5)	17 (65.4)	0.693	27 (61.4)	13 (65.0)	0.781
ASA grades (I/II/III), *n* (%)	16 (42.1)/17 (44.7)/5 (13.2)	7 (26.9)/11 (42.3)/8 (30.8)	0.186	20 (45.5)/17 (38.6)/7 (15.9)	3 (15.0)/11 (55.0)/6 (30.0)	0.057
Anesthetic methods (local/spinal/general), *n* (%)	2 (5.3)/25 (65.8)/11 (28.9)	2 (7.7)/7 (26.9)/17 (65.4)	0.009*	3 (6.8)/27 (61.4)/14 (31.8)	1 (5.0)/5 (25.0)/14 (70.0)	0.016*
Mesh repair (yes), *n* (%)	31 (81.6)	12 (46.2)	0.003*	11 (25.0)	10 (50.0)	0.028*
Operation time (min), mean ± SD	96.3 ± 26.3	105.1 ± 36.1	0.300	92.9 ± 24.3	115.2 ± 40.5	0.053

*M* median value, *CI* confidential intervals, *SD* standard deviation, *COPD* chronic obstructive pulmonary disease, *ASA* American society of anesthesia score

*Statistically significant

**Fig. 2:**
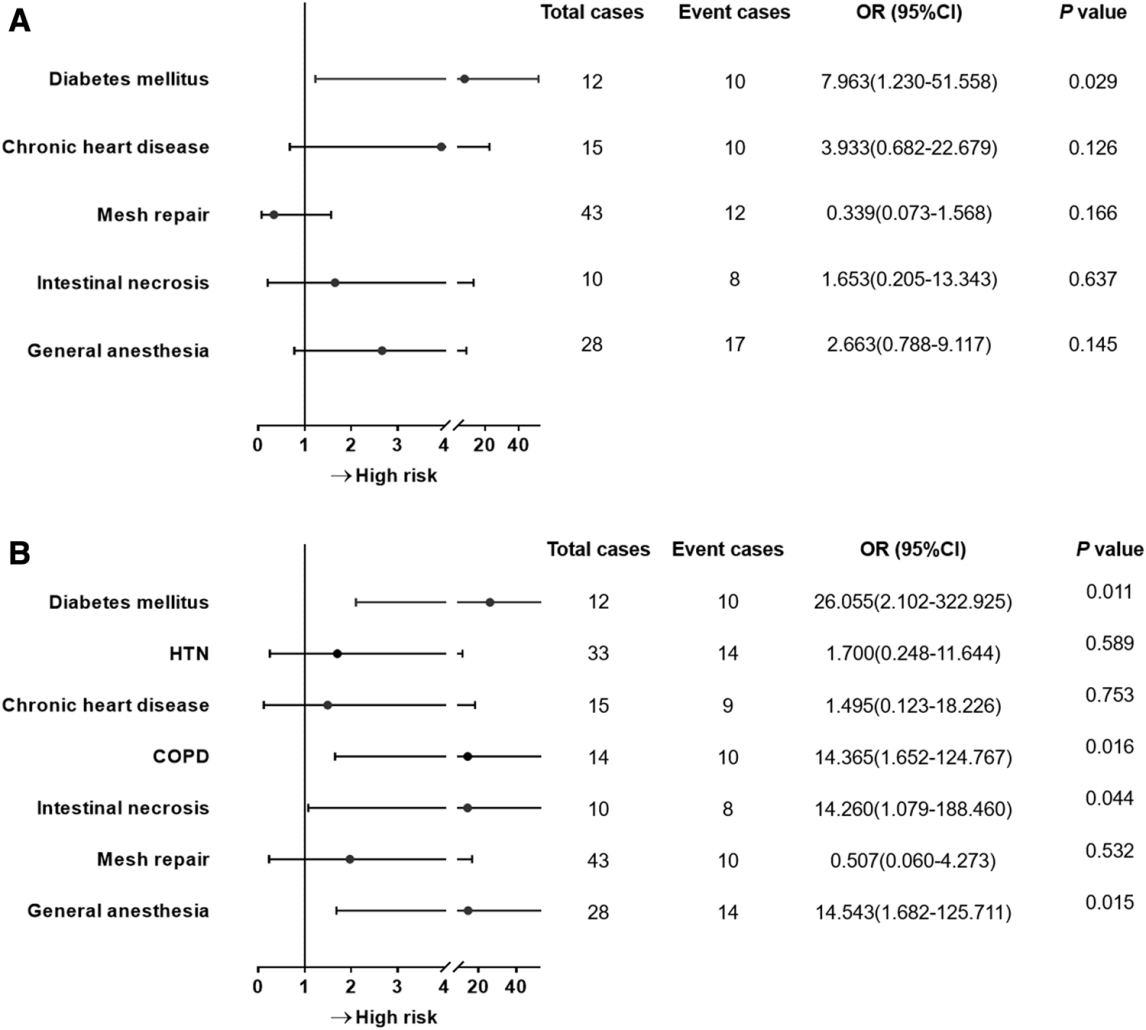
Multivariate logistic analysis of postoperative complications for incarcerated groin hernia. **a** Risk factors of postoperative complications; **b** risk factors of incisional complications after surgery. Factors that were statistically significant in univariate regression analysis were included in this stepwise logistic analysis. HTN, hypertension; COPD, chronic obstructive pulmonary disease; OR, odds ratio; CI, confidential interval. *P* < 0.05 denotes an independent risk factor of outcomes

The stepwise multivariate logistic analysis is summarized in Fig. 2. It showed that DM (*p* = 0.026) was an independent risk factor of postoperative complications, with DM (*p* = 0.011), COPD (*p* = 0.016), intestinal necrosis (*p* = 0.044) and general anesthesia (*p* = 0.015) as independent risk for incisional complications. Importantly, mesh repair was not independently associated with those complications for IGH.

## Discussion

In this study, the overall postoperative complication rate was 40.6%, with 31.2% and 6.2% for incisional complication rate and wound infection rate, respectively. Within the follow-up period, hernia recurrence and death were recorded in 7.8% of included patients each. Importantly, tension-free herniorrhaphy with PP mesh was performed in 67.2% of included subjects, and it was insignificantly associated with postoperative complications.

IGH in adult often involves bowel obstruction and potential necrosis, and it needs an emergency surgery to avoid death-related complications [13–15]. It is known that most of IGHs have the following characteristics: elder age, weakness due to concomitant disease, a long history of hernia with a large sac, and delayed diagnosis and treatment. Those features often lead to bowel necrosis or perforation, even severe complications such as diffuse peritonitis and sepsis, which are associated with increased rates of postoperative morbidity and mortality [3, 4, 16, 17]. A previous retrospective study indicates that long history of hernia, prolonged length of stay, severe concomitant diseases and high American Society of Anesthesia (ASA) grade were significant factors linked to unfavorable outcome of IGH [18].

Many studies have shown that the presence of concomitant diseases, such as DM, cardiovascular disorders, COPD and kidney disease, prolonged the length of stay and increased morbidity and mortality in IGH patients [2–4, 8, 16–19]. It is speculated that significant concomitant diseases greatly depressed oxygenation of surgical field, subsequently attenuated wound healing process and increased risk of wound complications after surgery [20, 21]. Additionally, numerous studies have confirmed that the presence of bowel necrosis or perforation made IGH patients more prone to bacterial translocation and wound infection after surgery [8, 10, 18, 19, 22–24]. In our study, DM was found to be an independent risk factor of postoperative complications, together with COPD, bowel necrosis and general anesthesia as independent risk factors for incisional complications. Our findings were in accordance with other studies [1, 6, 15–18, 22, 24].

Previous studies have reported that general or spinal anesthesia could increase the incidence of postoperative complications compared to local anesthesia [3, 8]. It is believed that local anesthesia has fewer adverse effects on respiratory function and provides better postoperative pain control in comparison with general anesthesia [25]. In clinical practice, selection of anesthetic technique is mainly determined by the severity of ASA class and intestinal incarceration. Consequently, general anesthesia was more frequently used in more severe cases, with poorer outcomes observed in comparison with regional or local anesthesia.

The application of synthetic mesh was limited in incarcerated hernia, especially suspicious of strangulated hernia [13, 26]. The conventional view suggested that using synthetic mesh would increase the chance of SSI and mesh-related complications [23, 27]. However, traditional tissue herniorrhaphy resulted in a high recurrence rate, and the requirement of additional repairing operation undoubtedly increased medical costs and frustrated patients. Recent studies have shown that it is safe and effective to use a prosthetic mesh in the emergency repair of incarcerated or strangulated hernia [1, 10, 28, 29]. Besides, it is recognized that intestinal necrosis and enterectomy are not absolute contraindications of performing a mesh repair [1, 2, 5, 7]. Moreover, some laparoscopic centers have reported that transabdominal preperitoneal repair can be safely applied for the management of incarcerated or strangulated hernia, with favorable outcomes achieved [26, 30].

In the current study, the overall incidence of postoperative complications in patients undergoing mesh repair was markedly lower than that in patients undergoing non-mesh repair (27.9% vs. 66.7%, *p* = 0.005), as well as the incidence of incisional seroma (20.9% vs. 47.6%, *p* = 0.028) and hernia recurrence (1.8–7%, *p* = 0.047). However, our results were predicated on patient selection bias by surgeons, with further studies required to obtain more powerful evidence-based data. Clinical utility of PP mesh can be considered when a hernia sac has been properly managed without residual contamination, and tension-free herniorrhaphy with such mesh has been confirmed to reduce recurrence and SSI rates [5, 10, 31].

As is well known, enterectomy is one of the worst complications of IGH, remaining an important risk factor of postoperative morbidity and mortality [4, 5, 10, 24]. It is reported that incarceration greater than 24 h, bowel perforation, severe hernia sac contamination and generalized peritonitis almost lead to an unavoidable enterectomy and make synthetic mesh repair unpracticable [22]. Under those conditions, use of biological mesh would be an alternative option, and several studies have shown its efficacy on potentially contaminated or infected herniorrhaphy [32].

This study had several limitations. First, the surgical strategy was made by surgeons’ preference, which came with an unavoidable selection bias. Tension-free herniorrhaphy was commonly selected for younger patients with shorter hernia duration and lower ASA grade. However, non-mesh tissue repair was applied for older patients with longer course, more significant concomitant illnesses, and bowel obstruction or necrosis. Besides, anesthetic regimen was often determined by the ASA class and personal experience of on-call anesthesiologists during surgery. Hence, general anesthesia was frequently applied in this cohort. Second, several confounding factors such as subjective factors of on-call surgeons and concrete concomitant diseases of these patients were hard to balance due to the small sample size of our cohort. At last, chronic pain degree and life of quality after surgery were not evaluated here due to its retrospective design. Other future studies with larger sample size and prospective study design must be required to illustrate the current findings.

## Conclusion

This study confirmed that DM was an independent risk factor of postoperative complications after emergency surgery for IGH, alongside with DM, COPD, intestinal necrosis and general anesthesia considered as risk factors for specific incisional complications. Mesh repair with various tension-free techniques could be safely and effectively performed for IGH patients, with better long-term outcomes obtained compared with non-mesh tissue repair.
